# Groin pain in athletes: a novel diagnostic approach

**DOI:** 10.1051/sicotj/2015017

**Published:** 2015-07-07

**Authors:** Vijay D. Shetty, Nikhil S. Shetty, Amith P. Shetty

**Affiliations:** 1 Hiranandani Orthopaedic Medical Education (HOME), Dr. L. H. Hiranandani Hospital Hillside Avenue, Hiranandani Gardens Powai, Mumbai 400076 India

**Keywords:** Groin pain, Athletes, Diagnosis, Femoroacetabular impingement, Hip arthroscopy

## Abstract

Groin pain in a performing athlete can be very challenging to diagnose and treat. The differential diagnosis includes intra-articular causes, extra-articular causes and non-musculoskeletal causes. A detailed clinical and radiological assessment of groin pain in this group is critical and can identify the underlying pathology. Diagnostic hip block is a valuable tool to differentiate intra-articular causes from extra-articular causes. Hip arthroscopy can help in identifying some of the elusive intra-articular conditions, which were once undiagnosed and therefore, left untreated, resulting in premature ending of competitive careers. This article attempts to explore current thinking on evaluation of groin pain, particularly in young individuals, and to establish a simple protocol for a clinical and diagnostic approach to this difficult problem.

## Introduction

Groin injuries account for 2% to 5% of all sports-related injuries with a high recurrence rate between 15% and 31% [1]. Inadequate evaluation of these injuries can result in premature ending of competitive careers [2]. Therefore, proper evaluation and appropriate treatment of groin pain particularly in a competitive athlete are paramount and can be very challenging [3–6]. Groin pain may result from an acute injury or repetitive trauma. Acute groin pain is commonly seen in sports that involve a sudden change in direction while running. In chronic groin pain, the ongoing complaints may be present for months to years [7].

There are no universally agreed guidelines on the clinical and diagnostic approach to recalcitrant groin pain. This article attempts to explore current thinking on evaluation of groin pain, particularly in young individuals, and to establish a simple protocol for a clinical and diagnostic approach to this difficult problem.

## Anatomy of groin pain

The groin consists of the area where the abdomen meets the legs and includes the structures of the perineum. The following structures comprise the groin: lower rectus abdominis musculature, inguinal region, symphysis pubis, upper portions of the adductor muscles of the thigh, the genitalia, as well as the scrotum in males.

From an anatomical point of view, various causes of groin pain can be considered under the headings of intra-articular and extra-articular causes [8, 9]. The intra-articular group consists of lesions arising within the ball and socket of the hip joint, while the extra-articular group includes conditions arising from outside the ball and socket joint [10]. Experts estimate that 60% of intra-articular injuries are initially misdiagnosed as extra-articular [11]. There have been a number of publications highlighting the presence and importance of these intra-articular injuries [12–14]. It is important to note that many conditions of non-musculoskeletal origin may have referred pain in the groin. These include gynaecological, urological, malignancies, sexually transmitted diseases and rheumatological conditions [15].

## Approach to groin pain in athletes

The approach to athlete with groin pain can challenge the clinician for a variety of reasons *as the cause of pain can be intra-articular*, *extra-articular or radiation* from elsewhere [16]. The first step in evaluating the groin pain in young athletes is to obtain a thorough history followed by a detailed physical examination to avoid missing the diagnosis. The key to *history taking* and the physical examination is to narrow down the differential diagnosis to either intra-articular pain or extra-articular pain.

A history of clicking on movement of the hip may indicate intra-articular pathology like labral tear, loose body or snapping hip. Byrd described the “C sign” in which a patient cups his or her hand above the greater trochanter in order to describe deep interior hip pain [8, 17]. Altered sensation or weakness together with a burning-type pain may indicate nerve entrapment. For example, obturator nerve entrapment is an uncommon condition, which causes pain in the region of the adductor muscles.

The physical assessment requires the exposure of as much of the groin and hip as permitted, in standing, supine and lateral position. A detailed examination of hip includes the following: inspection for anatomic irregularity; palpation of specific regions to localise tenderness [18]; assessment of the range of motion of the hip [19]; observation of the patient’s gait; and evaluation of performance activities like sprints and jumps which exacerbate the athlete’s pain.

Intra-articular pathology usually presents with painful restriction of movements of the hip. It is estimated that 27–90% of patients with groin pain have more than one coexisting injury [13]. Therefore it is possible for clinicians to diagnose and manage one injury, while a second injury may go unrecognised [20, 21]. Extra-articular causes such as osteitis pubis, muscle strain, avulsion injuries, stress fractures and nerve entrapment may be excluded by the type of onset pain and localising the site of tenderness. Acute and chronic causes of groin pain must also be differentiated. Acute injuries are relatively easy to diagnose based on history and physical findings, whereas chronic pain may be due to more than one cause and often requires additional diagnostic studies. A thorough physical assessment is required to exclude conditions causing referred pain in the groin *as in lumbar disc degeneration* [22].

## Imaging studies

**Figure 1.**
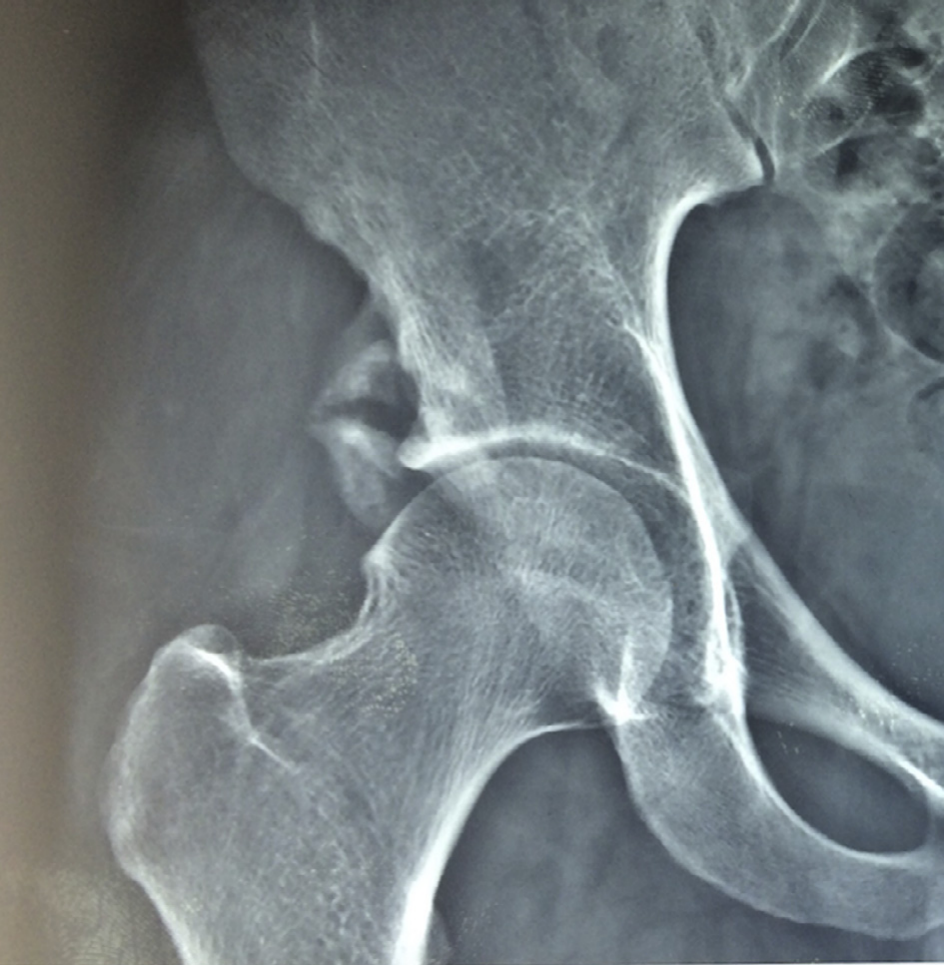
X-ray of right hip showing avulsion fracture of anterior inferior iliac spine in a young footballer.

**Figure 2.**
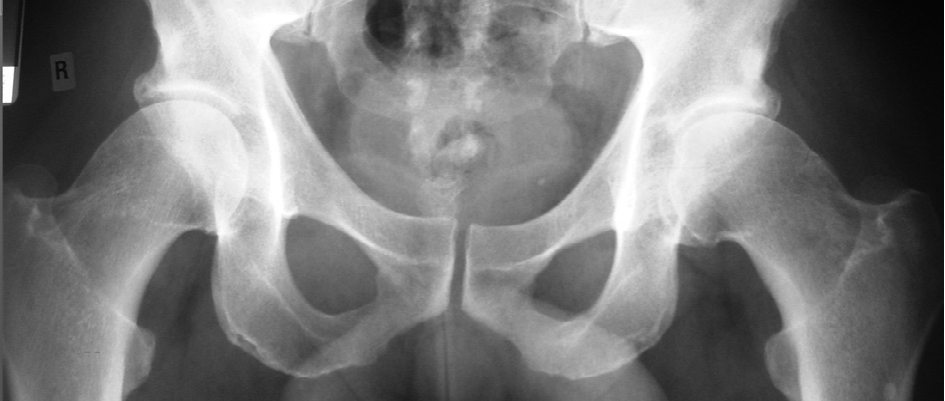
X-ray of the pelvis with both hips showing “pistol-grip” deformity of the proximal femur of both hips indicating “cam” type of impingement.

Plain radiograph of the hip remains the mainstay examination for most of the groin injuries (Figure 1). It remains as a baseline investigation, providing bony definition and alignment (Figure 2). Standardised radiographs are mandatory and should include the use of the correct film-focus distance and proper centring of the X-ray beam to prevent a false impression of altered joint morphology [23].

Ultrasound is a cheap, safe, quick and efficient tool providing exquisite soft tissue detail with the added advantage of providing a dynamic assessment. Dynamic ultrasonography seems to be a promising modality to exclude subtle hernias not detected by physical examination. Ultrasound has been reported to be also useful in diagnosing internal snapping hip syndrome (ISHS) caused by the iliopsoas tendon [16, 24, 25].

**Figure 3.**
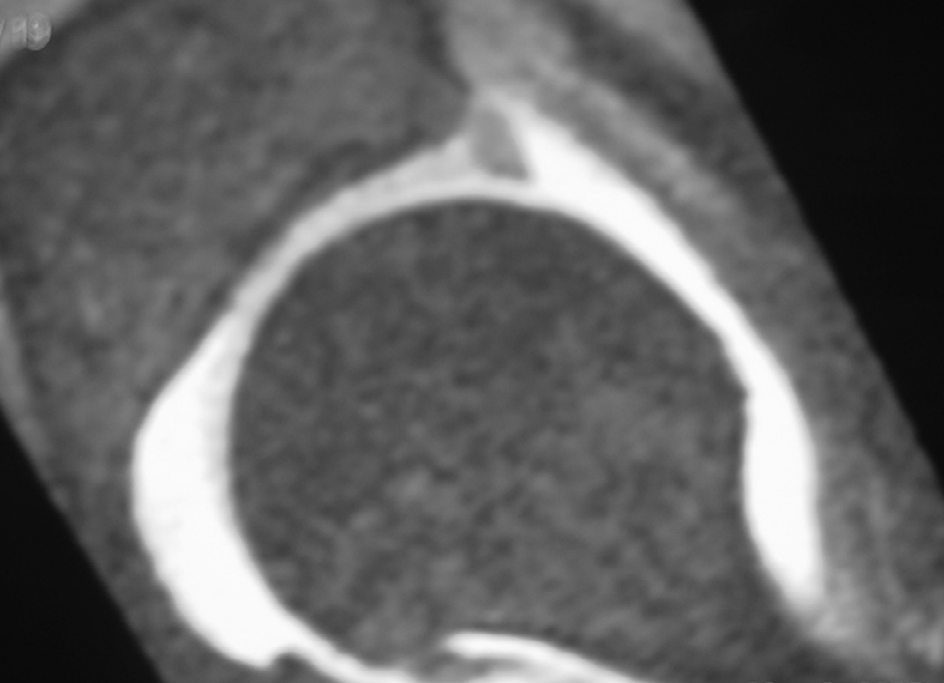
MRI arthrogram of left hip indicating acetabular labral tear.

Magnetic resonance imaging (MRI) is a powerful tool, providing excellent spatial resolution, and delineation of soft tissue structures including muscles, tendons and cartilage. It is a very sensitive and specific imaging modality for diagnosing osteochondral injuries, soft tissue injuries and inflammation. The use of contrast imaging can further increase the sensitivity of the examination due to enhancement of vascular lesions and in the assessment of avascular necrosis. Many authors advocate MRI combined with arthrography (MRA) of the hip for evaluation of labral pathology (Figure 3) and articular cartilage [26–28].

Multidetector computerised tomography (MDCT) provides excellent bony definition. MDCT 3D reconstruction images can help in surgical planning for bony procedures [29].

## Nerve conduction studies

Nerve conduction studies and electromyography (EMG) are very useful diagnostic tools for potential nerve entrapment syndromes, causing groin pain [30, 31].

## Diagnostic hip block

Fluoroscopic guided injections have proven to be an extremely valuable diagnostic and therapeutic test to localise groin pain of intra-articular origin [32, 33]. This may be done under general anaesthesia to ensure accurate intra-articular placement of the needle and should be followed by a carefully structured physiotherapy assessment in order to establish the intra-articular source of pain. Although the duration and extent of relief are variable, fluoroscopically guided, intra-articular injections of corticosteroid and local anaesthetic should typically alleviate symptoms attributable to labral tears, synovitis, mechanical impingement and osteoarthritic changes. A failed response to a well-placed injection should prompt evaluation for occult, extra-articular sources of symptoms [34]. A positive response to an intra-articular injection has been shown to be a 90% reliable indicator of an intra-articular abnormality [35]. *However*, *a negative response from an intra-articular hip injection may predict a higher likelihood of having a negative result from surgery* [36, 37]. The possibility of communication between the hip joint and iliopsoas bursa may exist and this needs to be assessed with MRI prior to the block [38].

## Hip arthroscopy

**Figure 4.**
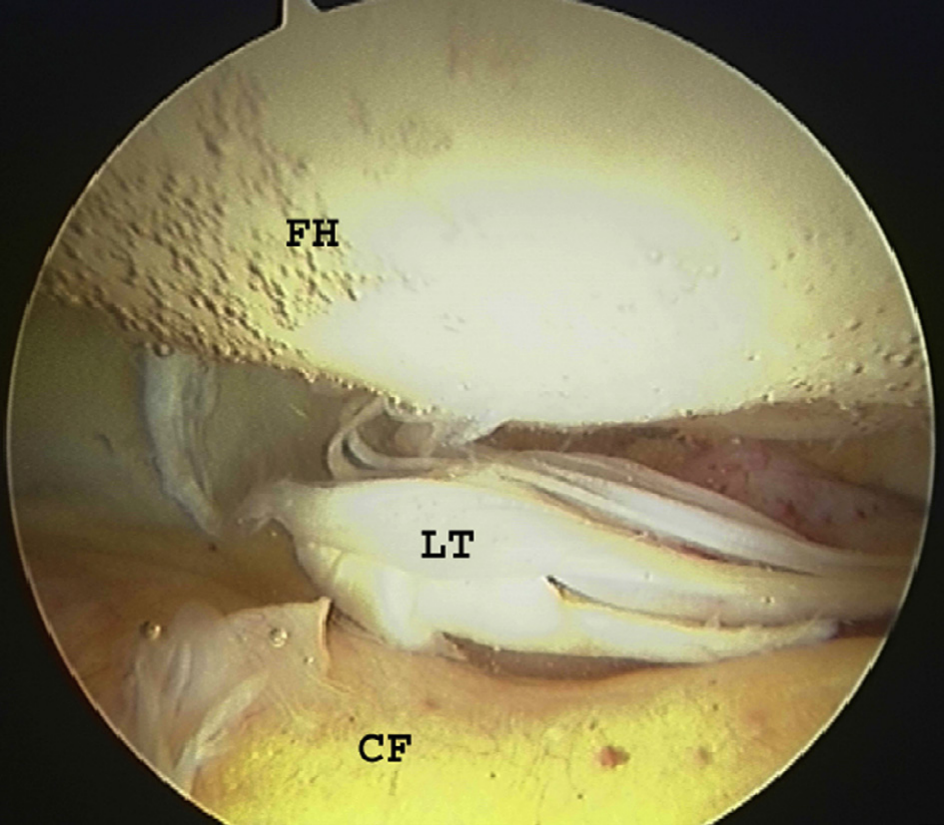
Arthroscopic view of the hip joint demonstrating ligamentum teres injury. FH = Femoral Head; LT = Ligamentum Teres; CF = Cotyloid Fossa. ©Richard Villar.

**Figure 5.**
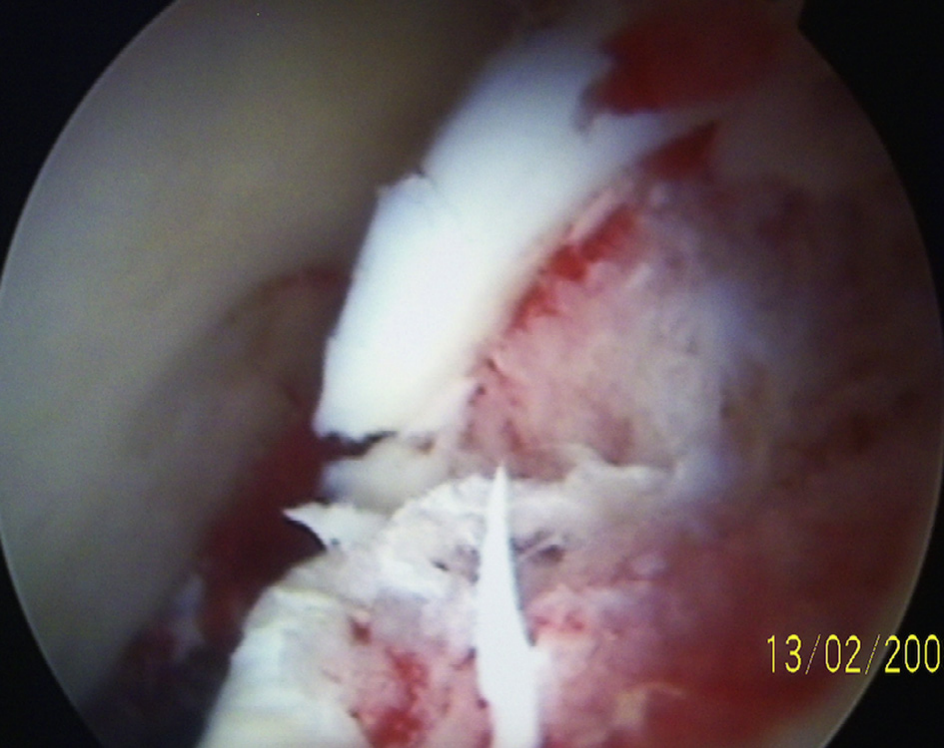
Arthroscopic view of the hip joint demonstrating severe chondral damage of the femoral head in a young man.

Recent years have seen increasing number of hip arthroscopies being performed worldwide. Hip arthroscopy is now a very well-established procedure [39–42]. This endoscopic procedure not only helps to diagnose some of these elusive intra-articular causes of groin pain (Figures 4 and 5) but also helps to treat specific lesions within the joint.

## Differential diagnosis for groin pain in athletes

**Figure 6.**
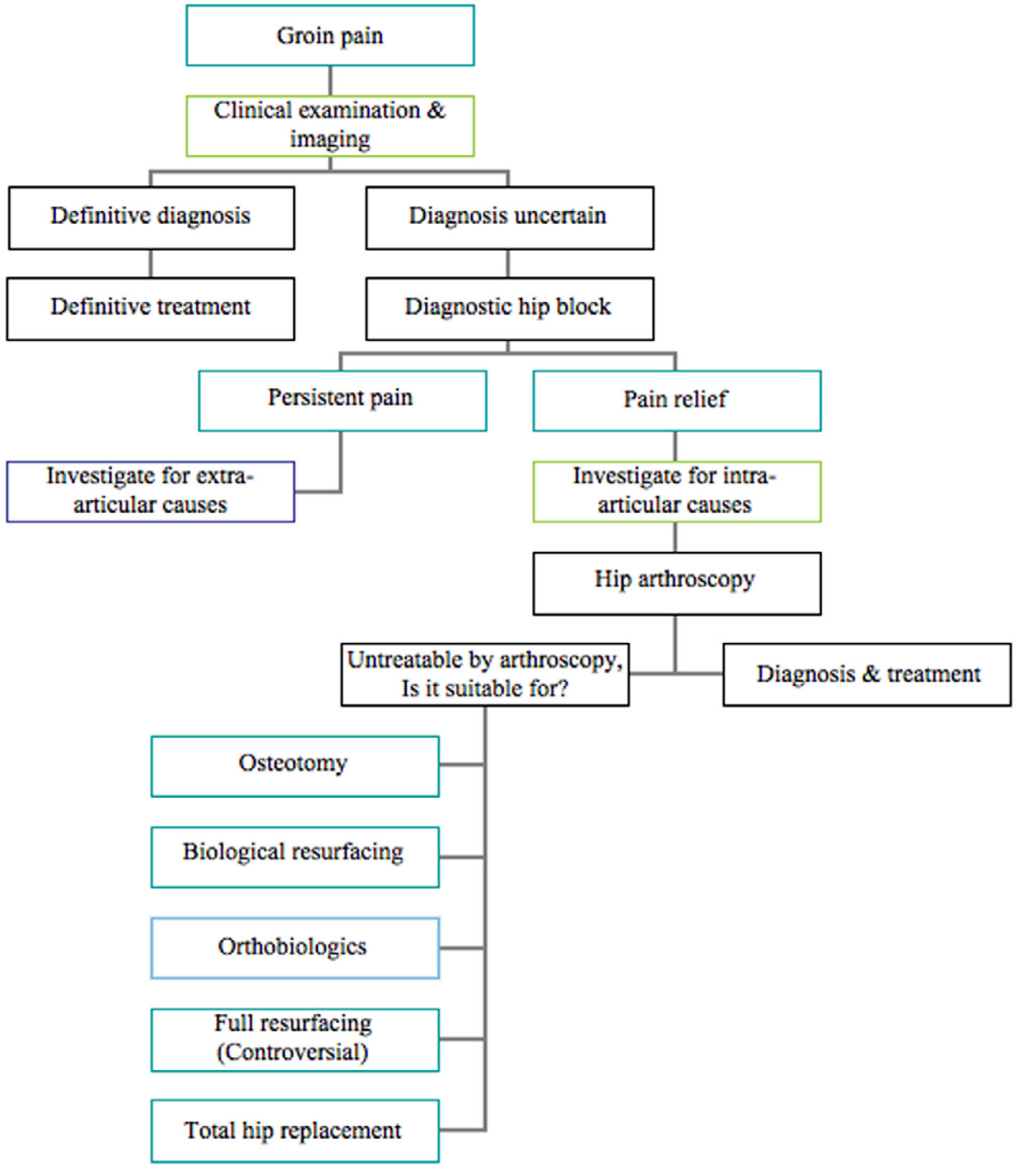
Our novel clinical approach to athletes with groin pain.

Table 1 shows the common intra-articular causes for groin pain in athletes with their clinical findings and related references. The common extra-articular causes for groin pain in athletes with their clinical findings and related references are outlined in Table 2. We follow a simple algorithm to approach recalcitrant groin pain in athletes as shown in Figure 6.

**Table 1.** Differential diagnosis for intra-articular causes of groin pain in athletes.

Common intra-articular pathologies causing groin pain			
	Conditions	Findings	Related references
1.	Femoroacetabular impingement (FAI)	Sharp anterior hip pain with deep flexion, internal rotation or abduction. Limited internal rotation and adduction in flexion. Positive impingement test.	[17, 30, 43–48]
2.	Chondrolabral injuries	Dull groin pain, worsens with activities like prolonged sitting, walking. Restricted terminal hip range of movements. Locking, clicking, giving way.	[41, 49–51]
3.	Injuries to the ligamentum teres	Hip stiffness. Giving way. Reduced range of motion.	[52–54]
4.	Loose bodies	Anterior groin pain. Catching, locking, clicking or giving way. Limited range of movements.	[55, 56]

**Table 2.** Differential diagnosis for extra-articular causes of groin pain in athletes.

Common extra-articular pathologies causing groin pain			
	Conditions	Findings	Related references
1.	Muscle strain/tears	Aching groin or medial thigh pain and may or may not relate a specific inciting incident. Painful restriction of movements especially adduction. Localised tenderness and focal swelling along adductors. Decreased adductor strength.	[57, 58]
2.	Stress fracture	Exercise induced pain in hip, groin, thigh or referred to knee that aggravates at night. Sudden worsening of groin pain suggests completion of fracture.	[59–63]
3.	Osteitis pubis	Anterior hip pain radiating to suprapubic area. Localised tenderness over pubic symphysis.	[57, 64, 65]
4.	Sports hernia	Insidious onset of groin pain on activity. Pain aggravates on sudden movements like coughing, sneezing, kicking and sprints.	[2, 15, 66, 67]
5.	Snapping syndromes	Groin pain that aggravates on movements. Intermittent catching, locking of hip.	[64]
6.	Nerve entrapment	Groin pain associated with burning sensation. Altered sensation along the distribution of nerve. Weakness of affected group of muscles.	[30, 68]

## Summary

Groin pain in a performing athlete can be very challenging to diagnose and treat. The differential diagnosis includes intra-articular causes, extra-articular causes and non-musculoskeletal causes. A detailed clinical and radiological assessment of groin pain in this group is critical and can identify the underlying pathology. Diagnostic hip block is a valuable tool to differentiate intra-articular causes from extra-articular causes. Hip arthroscopy can help in identifying some of the elusive intra-articular conditions, which were once undiagnosed and therefore, left untreated, resulting in premature ending of competitive careers.

## Conflict of interest

The authors declare that there is no conflict of interest regarding this article.
